# Pattern of **β**-Thalassemia and Other Haemoglobinopathies: A Cross-Sectional Study in Bangladesh

**DOI:** 10.5402/2012/659191

**Published:** 2012-06-14

**Authors:** M. Mesbah Uddin, Sharif Akteruzzaman, Taibur Rahman, A. K. M. Mahbub Hasan, Hossain Uddin Shekhar

**Affiliations:** Department of Biochemistry and Molecular Biology, University of Dhaka, Dhaka 1000, Bangladesh

## Abstract

Thalassemia and other structural haemoglobinopathies are the major erythrocyte formation disorder prevalent in certain parts of the world including Bangladesh. We investigated 600 cases of anaemic patients referred from various parts of the country for diagnosis and counselling during 3 months (April to June 2011) of time. The most common form of haemoglobin (Hb) formation disorder observed in 600 subjects studied was *β*-thalassemia minor (21.3%). Two other conditions, such as E-*β*-Thalassemia and HbE trait, were also fairly common (13.5 and 12.1%, resp.) in the total subjects studied. Other forms of haemoglobin formation disorders observed were HbE disease (9.2%), Hb D/S trait (0.7%), *β*-thalassemia major (0.5%), and *δ*-*β*-thalassemia (0.5%). The majority of the haemoglobinopathies belonged to neonatal to childhood period (0–15 years), followed by reproductive age group (16–45 years). Few old-age (46+ years) cases were also detected in course of clinical complications.

## 1. Introduction

Haemoglobinopathies are genetic defect that results in abnormal structure of one of the globin chains of the haemoglobin molecule. Defects in these genes can produce abnormal haemoglobin and anaemia. Abnormal haemoglobin appears in one of three basic conditions; structural defects in the haemoglobin molecule, diminished production of one of the two subunits of the haemoglobin molecule (thalassemias), and abnormal association of otherwise normal subunits. The patients suffering from beta-thalassemia major and HbE/beta-thalassemia do not survive for more than 5 years without blood transfusion [1]. More than 700 haemoglobin variants have been described that involve genes both from alpha and beta gene clusters. Occasionally, someone inherit two different variants from the alpha-globin gene cluster or two different variants from beta-globin gene cluster. This condition is called compound heterozygosity. The nature of two genes inherited determines whether a clinically significant disease state develops. Among these the thalassemias comprise a diverse group of disorders and are broadly classified into *α*, *β*, *δ*-*β* and *γ*-*δ*-*β* thalassemias, depending on the globin chain(s), which are insufficiently synthesized [2]. Since humans have 4*α*-globin genes on chromosome 16 and 2*β*-globin genes on chromosome 11, symptomatic *α*-thalassemia is rarer than *β*-thalassemia. In addition to the transfusion-dependent form of *β*-thalassemia, *β*-thalassemia major, there are milder conditions that may escape detection until adulthood. Because of their high frequency and severity, the *β*-thalassemias pose the most important public health problem.

Inherited disorders of haemoglobin synthesis are, therefore, an important cause of morbidity and mortality worldwide. They place a large burden to the patients, their families, and even their communities. They are generally not curable but can be prevented by population screening, genetic counseling, and prenatal diagnosis [3]. The present study was undertaken to evaluate the spectrum and pattern haemoglobinopathies in a selected population of Bangladesh.

## 2. Materials and Methods

### 2.1. Study Subjects

 A total of 600 individuals were recruited in this study. The samples were collected from different hospitals located at Dhaka City during April to June 2011. They were suspected of suffering from anaemia and referred to these hospitals from peripheral regions.

The samples were analyzed at the Department of Biochemistry and Molecular Biology, Dhaka University. Written informed consent was obtained from the study participants and local ethical review committee approved the design of the study.

### 2.2. Blood Samples

 About 2-3 mL intravenous blood samples were collected after obtaining informed consent using EDTA (ethylene diamine tetra acetic acid) as anticoagulants by disposable syringes and needles from each individual free of blood transfusions.

### 2.3. Haematological Analysis

The Sysmex XE-2100 system Haematology analyzer (Sysmex Corporation, Kobe, Japan) was used to determine peripheral cell count and red blood cell indices (RBC, Hb%, HCT, MCV, MCH, and MCHC) using standard procedure [4] that employed RF/DC detection method, hydrodynamic focusing, flow cytometry method and SLS-haemoglobin method.

### 2.4. Haemoglobin Electrophoresis

 Haemoglobin electrophoresis was carried out on agarose gel using the Hydragel K20 System (Sebia, Issy-les-Moulineaux, France). The resulting electropherograms were evaluated visually for pattern abnormality. Scanning densitometry was used to determine the relative concentration of individual haemoglobin fraction.

### 2.5. Statistical Analysis

 Statistical analysis was carried out using SPSS statistical package (version 11.5). Analysis of variance (ANOVA) of the data was used to detect overall difference in group means. Differences among group means were assessed using least significant difference (LSD).

## 3. Results

Out of 600 cases, 253 (42.2%) cases were found normal and 347 (57.8%) had one or the other form of haemoglobinopathies. Out of 347 abnormal cases, 174 (50.1%) were males and 173 (49.8%) were females, which gives an equal incidence in both males and females.

**Figure 1:**
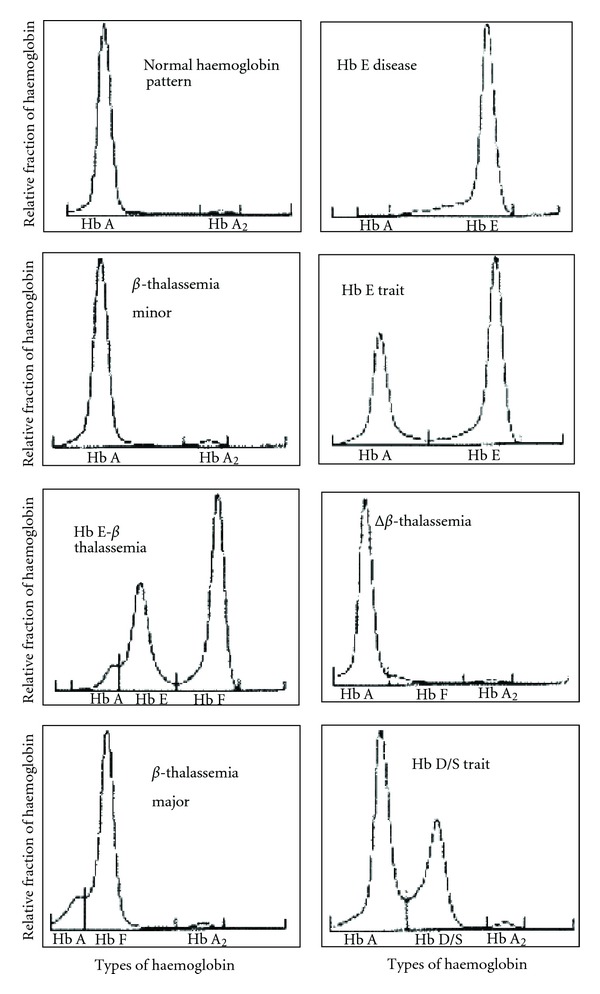
Electropherograms showing different types of haemoglobinopathies.

Table 1 represents the spectrum of haemoglobinopathies encountered during three months of time and Figure 1 depicted the electropherograms of different types of haemoglobinopathies. It is important to note here that *β*-thalassemia minor is the most common form of haemoglobinopathy (21.3%), followed by E- *β*-thalassemia (13.5%), HbE trait (12.1%), HbE disease (9.2%), Hb D/S trait (0.7%), *β*-thalassemia major (0.5%), and *δ*-*β*-thalassemia (0.5%), respectively. Though the study was conducted in Dhaka City, this data represent an overall picture of the country, because most of the patients were referred from peripheral regions, where such investigation facility is not available. 

Table 2 represents an age- and sex-wise distribution of cases of different haemoglobinopathies. It is apparent from the table that majority of the cases of haemoglobinopathy belong to neonatal to childhood period (0–15 years) followed by reproductive age group (16–45 years) and only a few cases of old age (46+ years) who were detected when they face clinical complications.

As shown in Table 3, patients of *β*-thalassemia minor had milder anemia with decreased total Hb (*P* < 0.001) and HbA (*P* < 0.001) and a slight increase in HbA_2_, E, and F compared to normal subjects. E- *β*-thalassemia patients suffered severe anaemia with significant decrease in total Hb (*P* < 0.001) and HbA (*P* < 0.001). There was significant increase in HbE (*P* < 0.001) and F (*P* < 0.001) even compared to *β*-thalassemia minor group with hypochromia and microcytosis. Patients with HbE disease suffered anaemia of same magnitude as E-*β*-thalassemia patients. The elevation of HbE and F was higher in this group than *β*-thalassemia minor group but lower than that of E-*β*-thalassemia group. In HbE-trait there was no sign of anaemia, still there was a significant increase in HbE (*P* < 0.001) level as found in HbE disease compared to *β*-thalassemia minor group. *β*-Thalassemia major patients had severe anaemia with significant decrease in total Hb and RBC count and showed highest increase in HbF levels among all the groups studied. *δ*-*β*-thalassemia and D/S trait groups showed milder anaemia with slight increase in HbA_2_ and HbF levels. However, *δ*-*β*-thalassemia group did not show an increase in HbE levels as it was evident in D/S trait group.

## 4. Discussion

Haemoglobinopathies are monogenic disorders of erythrocyte formation that has a widespread prevalence extending from Mediterranean zone, Middle East, Indian subcontinent, and parts of Southeast Asia [5]. Though the exact data regarding the prevalence and spectrum of haemoglobinopathies in Bangladesh is not known, it seems to be increasing. If the disease continues to pass vertically, it may take the form of epidemic. Since there is no effective treatment of the disease, the only way to prevent the disease is carrier detection and awareness among the people about this emerging epidemic. This study, therefore, provides a comprehensive data on the pattern of spectrum of haemoglobinopathies in Bangladesh.

Present study was conducted on 600 individuals referred to different hospitals at Dhaka City between April to June 2011. Among the 600 cases studied, 42.2% were found to be normal and 57.8% had one or the other forms of anaemia (Table 1). Out of 347 abnormal cases equal incidence in males (50.1%) and females (49.8%) was observed (Table 2). Most common haemoglobinopathies observed were *β*-thalassemia minor (21.3%), E-*β*-thalassemia (13.5%), haemoglobin E disease (9.2%), and haemoglobin E trait (12.1%). Several other studies also have documented such high frequency of these haemoglobin formation disorders in neighbouring and other Southeast Asian countries [6–9]. Other less common haemoglobinopathies observed were *δ*-*β*-thalassemia (0.5%), *β*-thalassemia (0.5%), and haemoglobin D/S trait (0.7%). The onset of the diseases was most prominent in neonatal to childhood period (0–15 years), followed by reproductive age group (16–45 years). Few cases of old age (46+ years) were detected only when they faced clinical complications (Table 2).

This study found many variations in the clinical presentation of disease (Table 3). Patients belonging to *β*-thalassemia minor group had mild anaemia with decreased total Hb (*P* = 0.001) and Hb A (*P* = 0.001) compared to normal. Haemoglobin electrophoresis showed a slight increase in HbA_2_ and F. Since these patients inherit one gene of *β*-thalassemia, they are either asymptomatic or develop mild-to-moderate anaemia. Though sometimes the red blood cells may be hypochromic and microcytic, iron supplementation is not required except for pregnant woman. Genetic counselling for couples at risk for offspring with homozygous *β*-thalassemia may be done at this stage.

Hb E-*β*-thalassemia was detected in 13.5% of the overall study population. Patients belonging to this group were severely anaemic with a significant decrease in total Hb (*P* = 0.001) and Hb A levels (*P* = 0.001). E-*β*-thalassemia patients inherit one gene for *β*-thalassemia and one gene for Hb E disease. As a result their clinical presentation is similar to those of *β*-thalassemia major or HbE disease, except that they showed greater elevation in HbE and F among all the groups studied. Many other studies also reported E-*β*-thalassemia as the commonest form of thalassemia in southeast Asia [6, 10, 11].

HbE disease and E trait are homozygotic and heterozygotic conditions of a *β*-chain variant, where a lysine residue is substituted by glutamic acid. This monogenic disease also has a broad distribution throughout the Mediterranean, the Middle East, and the Indian subcontinent [12, 13]. This study found 9.2% Hb E disease and 12.1% Hb E trait among the overall study subjects. The haematological parameters in HbE disease were similar with *β*-thalassemia major with the exception that *β*-thalassemic patients had a very high level of Hb F and absence of Hb E. Hb E-trait patients had milder anaemia comparable to *β*-thallasemia minor with significantly higher levels of Hb E (*P* = 0.001).

In *β*-thalassemia major, as expected, there was severe anaemia with extremely low total Hb with hypochromia and microcytosis. There was also a significant decrease in HbA and increase in Hb F levels. This finding is consistent with a different study by Pootrakul et al. [14]. *δ*-*β*-Thalassemia is similar to *β*-thalassemia major, but symptoms are milder. This study found only a slight increase in Hb F level still, much less compared to *β*-thalassemia major patients. The gene controlling delta chain production is located very close to beta gene on chromosome 11. If one gene is deleted then the other may be affected. Most of the patients of *δ*-*β*-thalassemia survive to adult life with minimal transfusion requirement.

Hb D/S trait is a heterozygotic condition where either one Hb D or S chain is combined with one normal *β* chain. Both D and S are variants of beta chain, in which a single amino acid is replaced. In Hb S the beta subunit has the amino acid valine at position 6 instead of glutamic acid, whereas in Hb D glutamine replaces glutamic acid at 121 positions on beta chain. Sickle cell disease is most common in people of African ancestry and tribal people of India. The carrier frequency of sickle gene is cited 1 in 10 in the USA and may be higher in Canada where the black population is composed largely of individuals of Caribbean and African origin [15]. Hb D on the other hand occurs mainly in northwest India, Pakistan, and Iran [16]. The frequency of Hb D/S trait found in this study was 0.7%, higher than *δ*-*β*-thalassemia, or *β*-thalassemia major. The electrophoretic mobility of Hb D and Hb S is identical at alkaline pH in cellulose acetate. So, this study could not differentiate them. However, they can be distinguished on citrate-agar electrophoresis at pH 6.2 [17].

## 5. Conclusion

*β*-thalassemia minor (21.3%) is the commonest encountered haemoglobin formation disorders followed by E-*β*-thalassemia (13.5%), HbE trait (12.1%), and HbE disease (9.2%) in Bangladesh. Since these patients were referred from peripheral regions of the country where the diagnostic facility is not much available, this data is representative of an overall prevalence of haemoglobinopathies and thalassemias of the country. An effective strategy of preventing the progression of the disease in Bangladesh might be a nation-wide screening program employing more sophisticated techniques like polymerase chain reaction (PCR) followed by direct sequencing, genetic counseling, and creating public awareness. 

## Figures and Tables

**Table 1:** Spectrum of haemoglobinopathies in Bangladesh.

Type of haemoglobinopathies	Incidence no. (%)		
	Male	Female	Total
Normal	166 (48.8)	87 (33.5)	253 (42.2)
*β*-Thalassemia	57 (16.8)	71 (27.3)	128 (21.3)
E-*β*-Thalassemia	48 (14.1)	33 (12.7)	81 (13.5)
Haemoglobin E disease	25 (7.3)	30 (11.5)	55 (9.2)
Haemoglobin E trait	38 (11.2)	35 (13.4)	73 (12.1)
*δ*-*β*-Thalassemia	1 (0.3)	2 (0.8)	3 (0.5)
*β*-Thalassemia major	2 (0.6)	1 (0.4)	3 (0.5)
Haemoglobin D/S trait	3 (0.9)	1 (0.4)	4 (0.7)

Total	340 (100.0)	260 (100.0)	600 (100.0)

**Table 2:** Age- and sex-wise distribution of cases of different haemoglobinopathies.

Age group (Years)	Incidence no. (%)		
	Male	Female	Total
0–15	112 (64.4)	81 (46.8)	193 (55.7)
16–45	54 (31.0)	82 (47.4)	136 (39.1)
46+	8 (4.6)	10 (5.8)	18 (5.2)

Total	174 (100.0)	173 (100.0)	347 (100.0)

**Table 3:** Haematological features of different types of haemoglobinopathies in the study subjects.

Variable	Normal	*β*-thalassemia minor	E-*β*-thalassemia	E-disease	E-trait	*β*-thalassemia major	*δ*-*β*-Thalassemia	D/S Trait	*P* value (for overall difference)
*N*	253	128	81	65	73	3	3	4	
Hb (g/dL)	12.3 ± 2.96	9.23 ± 2.06	6.12 ± 1.61	7.65 ± 3.76	10.9 ± 3.76	5.26 ± 1.30	11.3 ± 1.69	9.97 ± 5.00	<0.001
Hb A (%)	75.9 ± 17.7	60.4 ± 14.6	12.6 ± 5.32	2.39 ± 0.83	2.39 ± 0.84	12.2 ± 2.98	87.5 ± 3.32	53.1 ± 13.2	<0.001
Hb A2 (%)	2.02 ± 1.06	3.13 ± 1.26	—	—	—	—	2.70 ± 0.84	1.62 ± 0.21	<0.001
Hb E (%)	—	0.73 ± 0.07	45.6 ± 13.9	27.1 ± 4.41	30.2 ± 11.2	—	—	18.9 ± 7.72	<0.001
Hb F (%)	—	3.68 ± 1.35	41.5 ± 14.8	3.84 ± 1.60	0.15 ± 0.04	85.6 ± 3.75	9.75 ± 2.41	26.2 ± 7.32	<0.001
RBC (10^6^/*μ*L)	4.51 ± 0.84	4.77 ± 1.17	3.44 ± 0.87	3.92 ± 1.06	4.77 ± 0.67	2.80 ± 0.63	3.63 ± 0.12	4.49 ± 1.05	<0.001
HCT (%)	37.5 ± 7.82	30.7 ± 6.18	22.1 ± 5.08	24.9 ± 6.52	32.6 ± 4.73	18.6 ± 5.50	34.2 ± 1.97	31.4 ± 11.4	<0.001
MCV (fl)	79.4 ± 11.9	64.7 ± 12.4	64.8 ± 7.13	64.2 ± 7.37	68.7 ± 7.26	65.7 ± 4.85	82.0 ± 8.69	69.2 ± 13.8	<0.001
MCH (pg)	23.2 ± 6.12	19.5 ± 3.31	17.8 ± 2.13	18.3 ± 2.95	21.3 ± 3.17	18.7 ± 0.61	26.3 ± 1.20	21.6 ± 6.98	<0.001
MCHC (g/dL)	29.7 ± 5.40	31.7 ± 8.15	27.5 ± 2.25	28.6 ± 3.63	30.9 ± 2.57	28.5 ± 1.55	32.2 ± 1.97	30.6 ± 4.35	>0.05

Data are mean ± SD.
